# General practitioners’ experiences with, views of, and attitudes towards, general practice-based pharmacists: a cross-sectional survey

**DOI:** 10.1186/s12875-021-01607-5

**Published:** 2022-01-14

**Authors:** Ameerah S. Hasan Ibrahim, Heather E. Barry, Carmel M. Hughes

**Affiliations:** Primary Care Research Group, School of Pharmacy, Medical Biology Centre, Queen’s University Belfast, 97 Lisburn Road, Belfast, Northern Ireland BT9 7BL UK

**Keywords:** General practice, General practitioners, Primary health care, Pharmacists, Cross-sectional

## Abstract

**Background:**

There is limited United Kingdom (UK) literature on general practice-based pharmacists’ (PBPs’) role evolution and few studies have explored general practitioners’ (GPs’) experiences on pharmacist integration into general practice. Therefore, this study aimed to investigate GPs’ experiences with, views of, and attitudes towards PBPs in Northern Ireland (NI).

**Methods:**

A paper-based self-administered questionnaire comprising four sections was mailed in 2019 to 329 general practices across NI and was completed by one GP in every practice who had most contact with the PBP. Descriptive analyses were used and responses to open-ended questions were analysed thematically.

**Results:**

The response rate was 61.7% (203/329). There was at least one PBP per general practice. All GPs had face-to-face meetings with PBPs, with three-quarters (78.7%, *n* = 159) meeting with the PBP more than once a week. Approximately two-thirds of GPs (62.4%, *n* = 126) reported that PBPs were qualified as independent prescribers, and 76.2% of these (*n* = 96/126) indicated that prescribers were currently prescribing for patients. The majority of GPs reported that PBPs always/very often had the required clinical skills (83.6%, *n* = 162) and knowledge (87.0%, *n* = 167) to provide safe and effective care for patients. However, 31.1% (*n* = 61) stated that PBPs only sometimes had the confidence to make clinical decisions. The majority of GPs (> 85%) displayed largely positive attitudes towards collaboration with PBPs. Most GPs agreed/strongly agreed that PBPs will have a positive impact on patient outcomes (95.0%, *n* = 192) and can provide a better link between general practices and community pharmacists (96.1%, *n* = 194). However, 24.8% of GPs (*n* = 50) were unclear if the PBP role moved community pharmacists to the periphery of the primary care team. An evaluation of the free-text comments indicated that GPs were in favour of more PBP sessions and full-time posts.

**Conclusion:**

Most GPs had positive views of, and attitudes towards, PBPs. The findings may have implications for future developments in order to extend integration of PBPs within general practice, including the enhancement of training in clinical skills and decision-making. Exploring PBPs’, community pharmacists’ and patients’ views of this role in general practice is required to corroborate study findings.

**Supplementary Information:**

The online version contains supplementary material available at 10.1186/s12875-021-01607-5.

## Background

Primary care has been defined as a “first-contact, accessible, continued, comprehensive and coordinated care” [1]. This care is provided by multidisciplinary teams including general practitioners (GPs), pharmacists, practice nurses, and other healthcare professionals (HCPs) such as dieticians and physiotherapists [1, 2]. Primary care in the United Kingdom (UK) faces unprecedented challenges due to the growing complexity of an ageing population and their care needs [3]. Ageing is often linked with an increased prevalence of multimorbidity (the presence of two or more chronic conditions) and polypharmacy (the concomitant use of four or more medicines), which increases the demand for primary care services and GPs [3]. Furthermore, primary care faces a workforce crisis arising from issues around recruitment and retention of GPs and practice nurses [3]. Therefore, one approach to alleviate some of the pressures within primary care has been the integration of pharmacists into general practices, known as general practice-based pharmacists (PBPs) [4].

In 2015, five-year PBP pilot schemes were launched in both England and Northern Ireland (NI) to integrate PBPs into general practice [4, 5]. A shortage of approximately 8000 GPs in England with a projected excess of 11,000–19,000 newly qualified pharmacists by 2040 encouraged National Health Service (NHS) England to launch a £15 m initiative in 2015. This initiative aimed to employ more than 490 PBPs across 658 general practices to provide more support in the management of long-term conditions and enhance the standard of care for patients [4, 6–8]. Further investment was announced in 2016 as NHS England planned to invest a further £100 m to recruit and train an additional 1500 pharmacists by 2020/2021 [7, 9]. In NI, the Department of Health allocated £17 m of funding to support PBPs in general practices across NI [10]. It is anticipated that there will be 300 whole time equivalent PBPs in post by the end of the pilot scheme in NI (2020/2021) [5].

PBPs, as qualified experts in medications with a variety of knowledge and skills, have been able to improve access to healthcare and reduce waiting times for appointments in general practice [11]. Furthermore, PBPs have delivered a range of activities that have been found to enhance patient outcomes (e.g. resolution of medication-related problems and improved prescription appropriateness) [12, 13]. These activities include medication review and medication reconciliation [11, 12, 14]. Moreover, if the PBP is qualified as an independent prescriber, they are able to prescribe in areas in which they are competent, and conduct chronic disease review clinics [11, 14]. They may also undertake administrative tasks such as clinical audit and prepare prescribing protocols [15].

Establishment of interprofessional collaborative working [16] between primary HCPs is essential to improve service delivery and patient outcomes [17, 18]. It is important to explore GPs’ attitudes towards collaboration with PBPs as this may affect the degree to which they collaborate with one another [19], and may contribute to the ongoing development of the role. As integration of PBPs into general practices is a new initiative, there will be barriers to successful interprofessional collaboration that will need to be overcome. An Australian study identified the views of GPs and pharmacists (i.e. community pharmacists and PBPs) on the integration of pharmacists into general practice and found several benefits such as improved collaboration and communication amongst the primary healthcare team [20]. This Australian study also identified barriers such as negative practitioner perceptions, and insufficient funding and infrastructure [20]. A Canadian study described the barriers and facilitators that the primary care teams (PBPs, GPs and nurse practitioners) experienced during pharmacist integration [21]. Barriers and facilitators existed around relationships, trust and respect, definition of pharmacist role, support, pharmacist personality and professional experience, presence and visibility of pharmacists, and resources [21].

Literature is now emerging which describes the views and experiences of HCPs with the integration of pharmacists into general practice [20–23]. Several studies stressed the need to collect more detailed information regarding the context of the PBP role as little is known about how PBPs affect the healthcare system, including patients and HCPs [24–26]. Moreover, there is limited UK literature on PBPs’ role evolution and few studies have explored GPs’ experiences on pharmacist integration into primary care practice to date; none have explored the views of GPs in NI, where there has been regional deployment of PBPs across practices. Furthermore, the findings of studies conducted in other parts of the UK may not be generalisable to NI where established services and funding mechanisms are different. As the main reason for PBP integration into general practices was to reduce pressure on general practice, no research has explored the views of NI GPs regarding the role of PBPs and how this may have affected their workload and delivery of primary care. Therefore, this quantitative cross-sectional study aimed to address this gap in the literature by investigating: 1) GPs’ experiences with PBPs, 2) their views about the PBP role and its impact upon patients, and 3) their attitudes towards collaboration with PBPs.

## Methods

### Study design, population, and setting

The study used a cross-sectional design. One GP, in each general practice in NI, who had the most contact with the PBP, was invited to participate in this study. General practices are independent, small businesses, often operating from their own premises [2]. The job role of a GP can be designated as partner (GP responsible for running the business side of the practice and employing staff), salaried (GP receiving a salary for a contracted number of hours worked), or locum (GP providing temporary staffing cover at any time). The Business Services Organisation (BSO) website (see Table 1) maintains an up-to-date database of general practice postal addresses in NI [29]. There were 329 GP practices, 1342 registered GPs (excluding locums), and more than 2 million registered patients in NI on 4th September 2019 [30]. General practices are provided funding from the Health and Social Care Board (HSCB) (see Table 1) based on the number and types of patients registered with them [2].

**Table 1:** Summary of the key features of a number of health care organisations in Northern Ireland [27, 28]

Health care organisations in NI	Description
Health and Social Care Trusts [27]	- Five Trusts (the Belfast, Northern, Southern, South Eastern and Western Trusts) together with the NI Ambulance Trust. - Administrative health organisations which are responsible for the management and administration of health and social care services on a geographical basis.
Health and Social Care Board [27]	- Organisation responsible for commissioning health services, performance management of the Health and Social Care Trusts and service improvement.
Business Services Organisation (BSO) [27]	- Organisation responsible for the delivery of a variety of commercial support and specialist professional services to the Health and Social Care sector. - The BSO website provides comprehensive resources for primary care, such as the COMPASS report which is a prescribing information summary that is issued quarterly for each GP practice to provide GPs with feedback on their prescribing [28].

In NI, there are 17 GP Federations (a group of general practices, forming an organisational entity and working together within their geographical area) which offer the PBP terms and conditions of employment and provide occupational maternity pay as well as a sick pay scheme [31, 32]. Working patterns will be determined by the GP Federation and must meet the business needs of the Federation [32]. The salary of PBPs in NI depends on their experience and qualifications [32]. There were 2715 pharmacists registered in NI in 2020 of whom 12% were working as PBPs [33, 34]. Many PBP positions in NI general practices have been filled by experienced community pharmacists [33, 35]**.**

### Questionnaire

A postal questionnaire was selected as the most efficient method of administration which facilitates data collection from a large sample of participants in a relatively short period of time compared to other survey methods [36, 37]. This method also requires less social interaction with respondents (i.e. self-completion), thus social desirability bias and interviewer bias are reduced [36, 37].

The questionnaire was developed by the research team members (AHI, CH, HB), following a comprehensive literature search regarding perceptions of various HCPs (e.g. GPs, PBPs, and community pharmacists) on the role of PBPs [20–23, 38]. The nature and style of questions and presentation of questionnaire were considered to help optimise the response rate [39]. The questionnaire (see Additional file 1) comprised four sections: (A) demographic information about the GP respondent and their working environment; (B) extent of GPs’ collaboration with PBPs; (C) GPs’ attitudes towards collaboration with PBPs determined through administration of the Attitudes Towards Collaboration Instrument for GPs (ATCI-GP); and (D) GPs’ views on the role of PBPs and their impact in primary care.

ATCI-GP (Section C) is a validated five-point Likert scale developed to measure GP attitudes towards GP-pharmacist collaboration [19]. Permission was granted from the authors of the ATCI-GP to use the scale and to substitute the word ‘pharmacist’ for ‘practice-based pharmacist/PBP’ throughout the scale’s statements.

The entire questionnaire was piloted with three academic GPs from the School of Medicine, Queen’s University Belfast who were similar to the population of interest. They completed the questionnaire by self-administration. They were asked for their comments and general feedback regarding the content and flow of the questionnaire and their responses were used to refine its content and layout (i.e. face validity) and to estimate the time taken for its completion. The pilot responses were not included in the final sample or analysis.

Questionnaires were mailed, on two occasions during September (first mailing) and October (second mailing to improve response rate) 2019 to the 329 general practices in NI, accompanied by a covering letter and a return pre-paid addressed envelope. The cover letter which accompanied the questionnaire was directed to the Lead/Senior GP in each general practice (responsible for quality improvement and primary care management in the general practice) and requested that the GP who had the most contact with the PBP in their practice should complete the questionnaire. If the practice did not have a PBP, the cover letter requested that the questionnaire was still completed by a GP in order to obtain views on the implementation of PBPs in general practice.

In the cover letter, participants were assured of the confidentiality and anonymity of the collected data. Consent was deemed to be implicit if GPs returned completed questionnaires. This consent process was approved by the School of Pharmacy Ethics Committee – see later) [40].

### Statistical analysis

All returned questionnaires were coded then descriptive analyses were conducted such as age and gender distribution within the sample. Responses to the ATCI-GP statements (Section C), and GPs’ views statements (Section D) on the role of PBPs and their impact upon patients were also analysed descriptively, by calculating the percentage of agreement or disagreement to each statement. Data entry was doubled checked manually by the researcher (AHI) to ensure the absence of any errors within the data. Where there were missing responses in a questionnaire, these were coded as missing and were omitted from the final analysis. SPSS version 26.0 [41] was used for all statistical analysis.

A broad approach was taken to analyse responses to open-ended questions [42]. The responses were read several times to achieve a general understanding, and grouped under broad categories as a means to summarise the main findings.

## Results

A total of 203 completed questionnaires were received following both mailings, providing a response rate of 61.7% (203/329).

### Demographic data

Table 2 presents non-identifiable demographic data about GPs and their working environment. Some demographic characteristics of the GPs (i.e. gender and age) were compared with those of the entire population of GPs in NI from data published on the BSO website (see Table 2) [29]. Almost 60% (57.4%, *n* = 116) of GPs were male, which was a slightly larger proportion compared to the GP population in NI (42.4%; *n* = 573). GPs had a mean age of 50.4 (SD ±8.6) years and there were slight differences in the age groups between the GP respondents and all GPs in NI (see Table 2). On average, GPs had obtained a Certificate of Completion of Training or equivalent (qualified as a GP) 23.6 (SD ± 9.4) years ago. The mean number of sessions spent by GPs in general practice per week was 7.1 (SD ± 1.5). There was approximately equal distribution of the responses across the location of general practices and the five Trust areas in NI in which the majority of GPs’ patients predominantly reside, indicating broad geographical distribution at practice level. Three-quarters (76.8%, *n* = 156) of GPs indicated that they worked in medium-sized practices based on list sizes (i.e. 3000–10,000 patients). There was at least one PBP per general practice. The respondents answered all the sections in the questionnaire as all GPs had a PBP working in their general practices at the time of the study.

**Table 2:** Demographic profile of GP respondents (*n* = 203) in Northern Ireland

	***Number of GP respondents*** (%)	***Number of NI GPs***^***a***^ (%)
**Gender**		
Female	85 (42.1)	778 (57.6)
Male	116 (57.4)	573 (42.4)
Prefer not to say	1 (0.5)	*
**Age (years)**		
25–39	28 (14.2)	456 (33.8)
40–44	24 (12.2)	239 (17.7)
45–49	26 (13.2)	179 (13.2)
50–54	44 (22.3)	188 (13.9)
55–59	51 (25.9)	184 (13.6)
≥ 60 years	24 (12.2)	102 (7.5)
**Average years since the GP respondent had obtained Certificate of Completion of Training (CCT) or equivalent (qualified as a GP) (± SD)**	23.6 (± 9.4)	*
**Average number of GPs’ sessions per week (± SD)**	7.1 (±1.5)	*
**Location of general practices**		
Rural	61 (31.0)	*
Suburban	66 (33.5)	*
Urban	70 (35.5)	*
**Trust area of Northern Ireland in which majority of GPs’ patients predominantly reside**		
Belfast	40 (20.0)	*
Northern	48 (24.0)	*
South Eastern	41 (20.5)	*
Southern	39 (19.5)	*
Western	32 (16.0)	*
**Size of general practices**		
Small (< 3000 patients)	17 (8.4)	*
Medium (3000–10,000 patients)	156 (76.8)	*
Large (> 10,000 patients)	30 (14.8)	*
**Other health and social care professionals working within general practices**		
General practitioner (GP) – Partner	197 (98.5)	*
General practitioner (GP) – Salaried	81 (44.5)	*
Practice-based pharmacist (PBP)	203 (100)	*
Practice nurse	194 (98.0)	*
Others	117 (91.4)	*

^a^ Data published quarterly (equating to July–September 2019, published on October 1st 2019) on the BSO website [29]

* Information unavailable

### Characteristics of practice-based pharmacists and general practitioners – practice-based pharmacists’ communication

Approximately two-thirds of GPs (64.5%, *n* = 129) reported that PBPs had been working within general practice for 2 years or less at the time of questionnaire completion. Just over 45% of GPs (45.4%, *n* = 84) indicated that PBPs provided four to six sessions in GPs’ practices per week. Almost two-thirds of GPs (62.4%, *n* = 126) reported that PBPs were qualified as independent prescribers, with 76.2% (*n* = 96/126) indicating that PBPs were currently prescribing for patients.

All GPs had face-to-face meetings with PBPs, with three-quarters (78.7%, *n* = 159) meeting with the PBP more than once a week (see Additional file 2 – Figure A). The main issues usually discussed during these meetings were: medication issues, medication review, prescribing issues, patient issues, transitions between care sectors (e.g. hospital discharge and outpatient letters), work issues, audit and COMPASS reports, practice/system/Federation level issues, and training for PBPs. GPs used more than one method to communicate with PBPs, but the majority (95.5%, *n* = 192) indicated that face-to-face was the most common and preferred approach (see Additional file 2 – Figures B and C). Moreover, the GPs listed the most common reasons for the GPs to communicate with the PBPs and for PBPs to communicate with the GPs, e.g. patient issues and prescribing queries (see Additional file 3).

Frequency of face-to-face contact between PBPs and patients varied (see Additional file 4 – Figure A), e.g. 20.7% of GPs (*n* = 41) reported that PBPs had daily contact with patients, and 14.1% of GPs (*n* = 28) reported that PBPs did not meet face-to-face with patients. Additionally, they revealed that the main issues usually discussed during these meetings were chronic disease management clinic issues, medication problems, patient education, and other topics such as flu vaccinations. Furthermore, the majority of GPs (93.0%, *n* = 187) reported that the most common and preferred method of communication between PBPs and patients was by telephone (see Additional file 4 – Figures B and C).

The majority of GPs (70.6%, *n* = 142) indicated that the consulting room was always/very often available for PBPs, while 19.9% of GPs (*n* = 40) indicated that this room was sometimes available to PBPs (see Additional file 5).

**Fig. 1:**
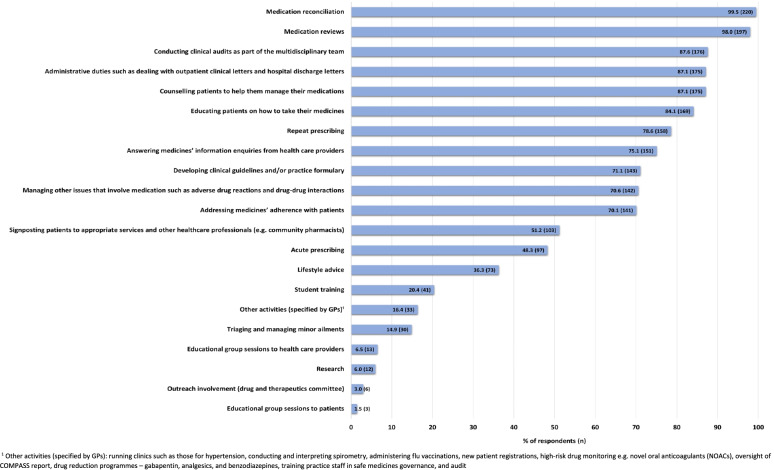
Activities of practice-based pharmacists in general practice as reported by responding general practitioners

PBPs provided a wide range of activities (see Fig. 1), most commonly medication reconciliation and medication reviews. Most GPs (92%, *n* = 185) noted that PBPs’ activities were allocated in general practice through mutual agreement between the GP and PBP. In addition, these activities were determined by the PBP’s current skills (63.7%, *n* = 128), the PBP’s level of confidence (55.2%, *n* = 111), and the PBP’s previous experience (54.2%, *n* = 109). Furthermore, 9.0% of GPs (*n* = 18) specified that PBPs’ activities were determined by: practice need, Federation demands, the HSCB’s plans [10], the lead PBP, or the PBP’s interests.

This survey investigated the frequency of issues encountered by GPs when dealing with PBPs (see Table 3). The majority of GPs reported that PBPs always/very often had the required clinical skills (83.6%, *n* = 162) and knowledge (87.0%, *n* = 167) to provide safe and effective care for patients and had the required experience to meet the needs of the practice (78.7%, *n* = 155). However, 31.1% (*n* = 61) stated that PBPs only sometimes had the confidence to make clinical decisions.

**Table 3:** Frequency of issues encountered by the general practitioners when dealing with practice-based pharmacists

Statement	Always *N* (%)	Very often *N* (%)	Sometimes *N* (%)	Rarely *N* (%)	Never *N* (%)
I do not have time to contact the PBP	1 (0.5)	13 (6.7)	48 (24.7)	74 (38.1)	58 (29.9)
The PBP struggles to adapt to the needs of the practice	1 (0.5)	5 (2.5)	28 (14.1)	69 (34.7)	96 (48.2)
The PBP has the clinical skills to provide safe and effective care for patients	81 (41.8)	81 (41.8)	25 (12.9)	6 (3.1)	1 (0.5)
The PBP has the required experience to meet the needs of the practice	75 (38.1)	80 (40.6)	36 (18.3)	6 (3.0)	0 (0)
The PBP is unavailable in the practice when I need them	4 (2.0)	29 (14.6)	77 (38.7)	62 (31.2)	27 (13.6)
The PBP has the confidence to make clinical decisions	35 (17.9)	82 (41.8)	61 (31.1)	15 (7.7)	3 (1.5)
The PBP has the knowledge to provide safe and effective care for patients	71 (37.0)	96 (50.0)	23 (12.0)	2 (1.0)	0 (0)
Patients are reluctant to accept and book an appointment with the PBP	1 (0.6)	1 (0.6)	53 (32.3)	81 (49.4)	28 (17.1)

Almost 40% of GPs (38.7%, *n* = 77) identified that sometimes, PBPs were unavailable in the practices when they were needed, and 32.3% (*n* = 53) stated that sometimes patients were reluctant to accept and book an appointment with the PBPs.

### Attitudes towards collaboration with practice-based pharmacists

Responses to the statements taken from the ATCI-GP (Section C) are summarised in Table 4. The majority of GPs (> 85%) agreed/strongly agreed with each of these statements, thereby displaying largely positive attitudes towards collaboration with PBPs.

**Table 4:** Attitudes of general practitioners towards collaboration with practice-based pharmacists

Statement	Strongly disagree/disagree *N* (%)	Neither agree nor disagree *N* (%)	Agree/strongly agree *N* (%)
1. The professional communication between myself and the PBP is open and honest	2 (1)	5 (2.5)	194 (96.5)
2. The PBP is open to working together with me on patients’ medication management	1 (0.5)	5 (2.5)	195 (97.0)
3. The PBP delivers high quality healthcare to patients	2 (1.0)	7 (3.5)	191 (95.5)
4. The PBP has time to discuss matters with me relating to patients’ medication regimens	7 (3.5)	16 (8.0)	178 (88.5)
5. The PBP meets the professional expectations I have of him/her	4 (2.0)	16 (8.0)	181 (90.1)
6. I can trust the PBP’s professional decisions	1 (0.5)	5 (2.5)	195 (97.0)
7. The PBP actively addresses patients’ medical concerns	5 (2.5)	18 (9.1)	175 (88.3)
8. The PBP and I have mutual respect for one another on a professional level	1 (0.5)	2 (1.0)	196 (98.5)
9. The PBP and I share common goals and objectives when caring for the patient	4 (2.0)	6 (3.0)	191 (95.1)
10. My role and the PBP’s role in patient care are clear	6 (3.0)	26 (12.9)	169 (84.1)
11. I have confidence in the PBP’s expertise in medicines and therapeutics	2 (1.0)	5 (2.5)	196 (96.6)
12. The PBP has a role in assuring medication safety (for example, to identify drug interactions, adverse reactions, contraindications etc.)	1 (0.5)	1 (0.5)	201 (99.0)
13. The PBP has a role in assuring medication effectiveness (for example, to ensure the patient receives the optimal drug at the optimal dose etc.)	1 (0.5)	11 (5.4)	191 (94.1)

### General practitioners’ views about the practice-based pharmacist role and its impact in primary care

In relation to the PBP role and its impact on primary care, the majority of GPs agreed/strongly agreed with many statements listed in Table 5 (notably statements 1, 3, 6, 7, 8 and 9). However, GPs had mixed views if the introduction of the PBP could remove roles from other members of practice teams (statement 4) and 24.8% of GPs (*n* = 50) were unclear if the PBP role moved community pharmacists to the periphery of the primary care team (statement 5).

**Table 5:** Views of general practitioners on practice-based pharmacists and their impact on primary care

Statement	Strongly disagree/disagree *N* (%)	Neither agree nor disagree *N* (%)	Agree/strongly agree *N* (%)
1. I welcome the PBP as part of the team	1 (0.5)	4 (2.0)	197 (97.5)
2. The role of the PBP is clear to me	5 (2.5)	26 (12.9)	171 (84.7)
3. I understand the difference between the roles of community pharmacists and PBPs	2 (1.0)	9 (4.5)	191 (94.6)
4. The introduction of the PBP role may take roles away from other members of the practice team	53 (26.2)	28 (13.9)	121 (59.9)
5. The introduction of the PBP role moves community pharmacists to the periphery of the primary care team	130 (64.4)	50 (24.8)	22 (10.9)
6. PBPs can provide a better link between general practices and community pharmacists	3 (1.5)	5 (2.5)	194 (96.1)
7. The introduction of the PBP role will have a positive impact on patient outcomes	3 (1.5)	7 (3.5)	192 (95.0)
8. PBPs will help in improving GPs’ knowledge and confidence about medications	7 (3.5)	14 (6.9)	181 (89.6)
9. PBPs will help to alleviate pressure within primary care	3 (1.5)	21 (10.4)	178 (88.1)
10. Having a PBP employed in general practices will save the NHS money by potentially freeing up GP time	15 (7.4)	36 (17.8)	151 (74.8)
11. Having a PBP employed in general practices will save the NHS money by reducing medicine waste	4 (2.0)	31 (15.3)	167 (82.7)

### Free text comments

More than half of GPs (59.1%, *n* = 120) provided free text comments at the end of the questionnaire. Most (*n* = 78) reported that they had positive experiences with PBPs and/or indicated the benefits of the PBP role to general practice and patient care.

*“The PBP is an extremely important and helpful addition to both patients, GPs, and nurses. It has helped to improve our medicines knowledge and undoubtedly improves safety”* (GP191)

An evaluation of the free text comments from those 120 GPs indicated several comments (see Additional file 6) such as GPs being in favour of more PBP input via more sessions, and more full-time posts. Furthermore, there were comments related to preference for working arrangements to be overseen by practices rather than Federations and further training for pharmacists in clinical skills.

## Discussion

### Summary

This study has revealed that the majority of GPs in our sample had positive views about the role of PBPs and positive attitudes towards collaboration with PBPs.

### Comparison with existing literature

All GPs had at least one PBP in their general practices, thereby highlighting the timeliness of the topic of this study. Three-quarters of GPs indicated that the PBP was currently prescribing for patients. This was a positive finding as the benefits of pharmacists being able to prescribe medicines has been highlighted previously [43, 44], such as better utilisation of pharmacists’ skills and knowledge, and enhancing patient care [44]. In contrast, less than 30% of GPs indicated that there were some PBPs currently not prescribing for patients which could limit their role in a general practice. In this context, PBPs could not implement changes and had to rely on a GP or another prescriber to address any recommendations [45]. Non-medical prescribers not actively prescribing has previously been reported in the literature [46, 47]. This has been attributed to a lack of financial support, lack of awareness of pharmacist prescribing by other HCPs, lack of access to patient clinical information [44, 47], and pharmacists lacking confidence in their ability as prescribers [48]. Furthermore, a lack of support from GPs and a lack of GP confidence in pharmacist prescriber abilities might be challenges encountered by pharmacists as they develop as prescribers [48].

All GPs had face-to-face meetings with PBPs. This is positive, as lack of direct communication has been identified as one of the challenges associated with the collaboration of two professions, particularly community pharmacists and GPs [49, 50]. As PBPs are sharing a workplace with GPs, this can enhance interprofessional communication and improve these relationships [24, 38, 51].

GPs indicated that PBPs were providing a wide range of activities. Medication reconciliation and medication reviews were a major part of PBPs’ role. Most of these activities have been reported previously, demonstrating that the activities of PBPs in NI were generally similar to those noted in the literature [12, 13, 52, 53]. Furthermore, over 90% of GPs reported that PBPs’ activities were determined through mutual agreement between the GP and PBP. This may reflect the presence of an established relationship and good communication between the GP and PBP.

Most GPs reported that PBPs always or very often had the clinical skills and the knowledge to provide safe and effective care for patients. This was reassuring as pressure on general practices is driven by an ageing population [3], the vast majority of whom are more likely to struggle with complex medication regimens that are associated with adverse events [54]. However, approximately a third of GPs stated that PBPs only sometimes had the confidence to make clinical decisions which may increase GPs’ workload; less confident PBPs may require reassurance and input from GPs on regular basis. This may reflect the novelty of this role and variation in previous experience that could limit PBPs’ confidence and ability to assume particular responsibilities [25].

Approximately 40% of GPs identified that sometimes the PBPs were unavailable in the practices when they were needed. Previous studies identified the limited time that pharmacists spent in the practice due to working part-time hours as a potential barrier to integrating pharmacists into general practices [20, 51, 55]. This finding highlights the importance of having a full-time PBP in the practice. Moreover, a third of GPs stated that sometimes patients were reluctant to accept and book an appointment with PBPs. This could be due to patients’ unfamiliarity with and lack of awareness of the PBP role [20, 51, 56]. Karampatakis et al. [57] explored patients’ experiences of PBPs and found that patients were unaware of pharmacists’ presence in general practice and/or unclear when to contact pharmacists. However, the study indicated that PBPs had the ability to improve the timely access to, and quality of, services in primary care. Furthermore, the study highlighted that there was a need to properly educate patients and the public about PBPs, including roles and responsibilities [57].

GPs’ attitudes towards collaboration were largely positive, suggesting the development of strong interprofessional collaboration and showing respect and trust between the two professions is essential [21, 38]. To develop this relationship, time, good communication, and effort on both parts are required [21, 58]. When PBPs work alongside GPs as part of a team, improvements in patient outcomes and greater patient satisfaction can be achieved [13, 59].

Almost all GPs welcomed the PBP as part of the primary care team. Previous studies have also noted positive GP views towards the integration of PBPs into general practice [20, 38, 52]. In this present study, the majority of GPs agreed/strongly agreed that the role of PBPs was clear to them. Understanding the role of the pharmacist by practice team members is essential in order to ensure their successful integration and utilisation of pharmacists’ skills and contributions [21, 51, 60]. Previous studies reported a lack of clarity on PBPs’ role and suggested the need for a clear definition [21, 51]. A recent qualitative study indicated that community pharmacists were aware of pharmacists’ presence in general practice but were uncertain about details such as employment models, roles and responsibilities. This highlights that there is a need to inform community pharmacists about PBPs’ scope of practice and to introduce formal regular meetings between community pharmacy and general practice staff [61].

Most GPs agreed/strongly agreed that PBPs could provide a better link between general practices and community pharmacists. In contrast, approximately 25% of GPs were unclear if the PBP role moved community pharmacists to the periphery of the primary care team. Other studies have reported mixed views from GPs and pharmacists on this topic, highlighting positive effects such as improving communication between GPs and community pharmacists [53, 61]. Potential negative effects may be role duplication or undermining the position of community pharmacists [20, 55]. Investigating the impact of PBP on the role of the community pharmacist would be important to ensure both branches of the profession can practise in a complementary manner for the benefit of patients and the profession.

### Strengths and limitations

A major strength of this study was its response rate of over 60% (61.7%; *n* = 203 GPs). This response rate was considered acceptable, based on recommendations in the literature [62]. Moreover, it was either higher than or comparable to the response rate obtained in postal surveys distributed in other studies in NI to GPs [63, 64], or to individual practices [65], or other cross-sectional studies published in primary care journals [66]. This response rate also indicated the topicality of this innovation in primary care, and the interest of the general practice profession. The method of administration and completion may also have reduced response bias.

Study limitations must be acknowledged. The study sample was limited to NI, and some findings may not be relevant to other parts of the UK. It is important to note that there were some differences between the key demographics of the responding GPs and the overall GP population in NI. Moreover, the study result may not be generalisable to GPs in NI who did not take part in this study as it is not possible to conclude that non-responding GPs would have held similar views. However, as noted, there was a high response rate and several findings were consistent with other international quantitative and qualitative studies on this topic [25, 51, 52, 67]. The questionnaire was not formally validated, however, the pilot phase was intended to address certain issues concerning face validity. Using an anonymous self-administered questionnaire which included both positive and negatively phrased items may have minimised the potential for social desirability bias [68].

### Implications for research and practice

The findings from the present study may have implications for future developments in order to extend integration of PBPs within general practice, including the enhancement of training in clinical skills and clinical decision-making. Moreover, an evaluation of the free-text comments indicated support for additional PBP input via more sessions and more full-time posts, different working arrangements, and development of further skills which may impact upon the successful integration of PBPs within general practice. As most GP respondents had positive views and attitudes regarding the role of PBPs and their impact in the primary care, this may encourage other countries to integrate pharmacists into general practice where there are health workforce shortages [69]. Aspects of the findings might also be useful to Australia, Canada and New Zealand, all of which have formal programmes for integrating and evaluating pharmacists’ services in general practice [70–72]. Importantly, future research should explore patients’ views and awareness of the role of the PBPs as well as their ability to differentiate between community pharmacists’ roles and PBPs’ roles. Additionally, further work is required to explore PBPs’ and community pharmacists’ views of this role in general practice to corroborate study findings.

## Conclusions

Most GPs in this cross-sectional survey highlighted that PBPs always/very often had the required clinical skills and the knowledge to provide safe and effective care for patients. However, a lack of confidence to make clinical decisions was noted and should be addressed to enhance integration of PBPs into general practices. The majority of GPs displayed largely positive attitudes towards collaboration with PBPs. Furthermore, most GPs had positive views about the PBP role, its impact in primary care and almost all GPs welcomed PBPs as part of practice teams. The findings may have implications for future developments in order to extend integration of PBPs within general practice and to add to the evidence base regarding PBPs’ impact in primary care.

## Supplementary Information

**Additional file 1.** General practitioner questionnaire. Description of data: Questionnaire that was distributed to general practitioners during this study.

**Additional file 2.** GP-PBP communication as reported by responding GPs. Description of data: Three figures (A, B, and C) depicting GP-PBP communication reported by GPs: Figure A shows frequency of face-to-face meetings between GPs and PBPs; Figure B shows the most common method(s) of communication between GPs and PBPs; and Figure C shows the most preferred method(s) of communication between GPs and PBPs.

**Additional file 3.** Most common reasons for GP-PBP communication as reported by responding GPs. Description of data: Table summarising the most common reasons for GP-PBP communication (with selected examples) reported by GPs.

**Additional file 4.** PBP-Patient communication as reported by responding GPs. Description of data: Three figures (A, B, and C) depicting PBP-Patient communication reported by GPs: Figure A shows frequency of face-to-face meetings between PBPs and patients; Figure B shows the most common method(s) of communication between PBPs and patients; and Figure C shows the most preferred method(s) of communication between PBPs and patients.

**Additional file 5.** Frequency of availability of a consulting room for use by the PBP. Description of data: Figure showing the frequency of availability of a consulting room for use by the PBP as reported by GPs.

**Additional file 6.** Recommendations and issues for the PBP role with supporting comments reported by GPs. Description of data: Table summarising the recommendations and issues that have been reported by GPs in the free text comments with examples of GP quotes.

## Data Availability

The data underlying this article will be shared on reasonable request to the corresponding author.

## Abbreviations

ATCI-GP: Attitudes Towards Collaboration Instrument for GPs
BSO: Business Services Organisation
GPs: General practitioners
HSCB: Health and Social Care Board
NHS: National Health Service
NI: Northern Ireland
PBP: General practice-based pharmacists
UK: United Kingdom
