# Supplementary material for: General practitioners’ experiences with, views of, and attitudes towards, general practice-based pharmacists: a cross-sectional survey
Source: BMC Prim Care. 2022 Jan 14;23:6. doi: 10.1186/s12875-021-01607-5 (PMC8759266; doi:10.1186/s12875-021-01607-5)
Supplement: Supplementary file 1 — Additional file 1. General practitioner questionnaire. Description of data: Questionnaire that was distributed to general practitioners during this study. [file 12875_2021_1607_MOESM1_ESM.docx]

**Additional file 1. GP questionnaire**


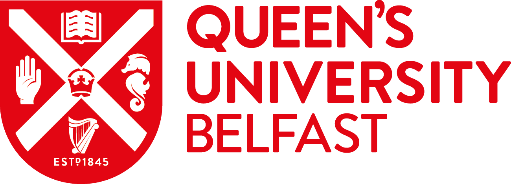


**AN EXPLORATION OF NORTHERN IRELAND GENERAL PRACTITIONERS’ EXPERIENCES WITH, VIEWS OF, AND ATTITUDES TOWARDS, PRACTICE-BASED PHARMACISTS**

| Section A | Demographic information |
| --- | --- |
| Section B | The extent of collaboration between GPs and practice-based pharmacists (PBPs) |
| Section C | Attitudes towards collaboration with practice-based pharmacists (PBPs) |
| Section D | Views on practice-based pharmacists (PBPs) and their impact in primary care |

**HOW TO COMPLETE THIS QUESTIONNAIRE:**

This questionnaire should take approximately 10-15 minutes to complete.

All the information gathered will be anonymous and cannot be linked to you as an individual.

There are no right or wrong answers and all answers are useful.

We are interested in your personal views, not what you think we want to hear.

**SECTION A: DEMOGRAPHIC INFORMATION**

*This section of the questionnaire is concerned with gathering some details about you and where you work.*

| 1. **What is your gender?** | | | | | | |
| --- | --- | --- | --- | --- | --- | --- |
| Female | | | Male | | Prefer not to say | |
| 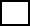 | | | 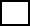 | | 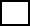 | |
| 1. **Please state your age in years:** | | | | | | |
| _______________________________________________________________________________ | | | | | | |
| 1. **In what year did you obtain your Certificate of Completion of Training (CCT) or equivalent?** | | | | | | |
| ________________________________________________________________________________ | | | | | | |
| 1. **How many sessions per week do you spend in clinical practice?** | | | | | | |
| ________________________________________________________________________________ | | | | | | |
| 1. **How would you describe the location of the general practice in which you currently work?** | | | | | | |
| Rural | | | Suburban | | Urban | |
| 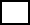 | | | 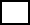 | | 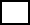 | |
| 1. **In which Trust area of Northern Ireland do the majority of your patients predominantly reside?** | | | | | | |
| Belfast | Northern | South Eastern | | Southern | | Western |
| 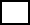 | 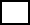 | 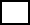 | | 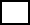 | | 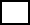 |
| 1. **How would you best describe the size of the general practice in which you currently work?** | | | | | | |
| Small  (<3,000 patients) | | | Medium  (3,000 – 10,000 patients) | | Large  (>10,000 patients) | |
| 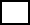 | | | 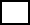 | | 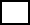 | |
| 1. **What other health and social care professionals work within your general practice?**   **Please circle yes or no. If you circle yes, then please indicate the number of other health and social care professionals in the space provided.** | | | | | | |
| **Health and social care professionals** | | | **Circle yes or no** | | **Number of health and social care professionals** | |
| General practitioner (GP) – Partner | | | Yes / No | | **______________** | |
| General practitioner (GP) – Salaried | | | Yes / No | | **______________** | |
| Practice-based pharmacist (PBP) | | | Yes / No | | **______________** | |
| Practice nurse | | | Yes / No | | **______________** | |
| Other  (Please specify below:) | | | Yes / No | | **______________** | |
| ________________________________________________________________________________________________________________________________________________________________________________________________________________________________________________________________________________________________________________________________________________________________________________________________________________ | | | | | | |
| **Please note: If there is no PBP in your general practice, go to Section D.** | | | | | | |

**SECTION B: COLLABORATION WITH PRACTICE-BASED PHARMACISTS**

*The following questions focus on your collaboration with practice-based pharmacists (PBP) to explore the extent of this collaboration.* ***Please note: If there is more than one PBP in your general practice, please answer the questions in this section based on the PBP with the most experience within your practice. If there is no PBP in your general practice, go to Section D.***

| 1. **How long has the current PBP worked in your general practice?** | | | | | | | | | | |
| --- | --- | --- | --- | --- | --- | --- | --- | --- | --- | --- |
| Less than 1 year | | | | 1 to 2 years | | | | More than 2 years | | |
| 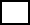 | | | | 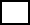 | | | | 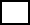 | | |
| 1. **How many sessions per week does the PBP spend in your general practice?** | | | | | | | | | | |
| ________________________________________________________________________________ | | | | | | | | | | |
| 1. **Is the PBP in your general practice qualified as an independent prescriber?** | | | | | | | | | | |
| Yes | | | No  (Go to Q13) | | | | Don’t know | | | |
| 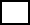 | | | 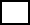 | | | | 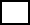 | | | |
| 1. **Is the PBP in your general practice currently prescribing for patients?** | | | | | | | | | | |
| Yes | | | No | | | | Don’t know | | | |
| 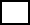 | | | 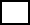 | | | | 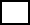 | | | |
| 1. **On average, how often do you meet face-to-face with the PBP?** | | | | | | | | | | |
| Never  (Go to Q15) | Daily | 2-3 times/ week | | | Once a week | Once a fortnight | | | Once a month | Other* |
|  |  |  | | |  |  | | |  |  |
| *Please specify below: | | | | | | | | | | |
| ________________________________________________________________________________ | | | | | | | | | | |

| 1. **If you meet face-to-face, what are the main issues that are usually discussed during these meetings?**   **Please list these issues below:** | | | | | |
| --- | --- | --- | --- | --- | --- |
| ________________________________________________________________________________________________________________________________________________________________  ________________________________________________________________________________  _______________________________________________________________________________  ________________________________________________________________________________ | | | | | |
| 1. **What is/are the most common method(s) of communication between yourself and the PBP?**   **Please tick all that apply:** | | | | | |
| Email | Face-to-face | Instant messaging | Telephone | Written | Other* |
| 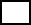 | 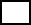 | 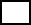 | 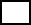 | 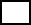 | 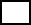 |
| *Please specify below: | | | | | |
| ________________________________________________________________________________ | | | | | |
| 1. **What is/are the most preferred method(s) of communication between yourself and the PBP?**   **Please tick all that apply:** | | | | | |
| Email | Face-to-face | Instant messaging | Telephone | Written | Other* |
| 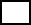 | 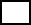 | 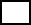 | 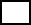 | 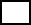 | 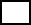 |
| *Please specify below: | | | | | |
| ________________________________________________________________________________ | | | | | |

| 1. **What are the most common reasons for you to communicate with the PBP?**   **Please list the reasons below:** | | | | | | | |
| --- | --- | --- | --- | --- | --- | --- | --- |
| ______________________________________________________________________________________  ______________________________________________________________________________________  **______________________________________________________________________________________** | | | | | | | |
| 1. **What are the most common reasons for the PBP to communicate with you?**   **Please list the reasons below:** | | | | | | | |
| __________________________________________________________________________________________________________________________________________________________________________________________________________________________________________________________________ | | | | | | | |
| 1. **On average, how often does the PBP have face-to-face contact with patients in your general practice?** | | | | | | | |
| Never  (Go to Q21) | Daily | 2-3 times/ week | Once a week | Once a fortnight | Once a month | Other* | Don’t know |
| 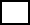 | 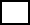 | 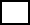 | 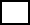 | 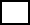 | 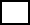 | 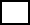 | 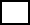 |
| *Please specify below: | | | | | | | |
| ______________________________________________________________________________________ | | | | | | | |
| 1. **What are the main issues usually discussed during face-to-face contact between the PBP and patients?**   **Please list these issues below:** | | | | | | | |
| ____________________________________________________________________________________________________________________________________________________________________________  ______________________________________________________________________________________ | | | | | | | |

| 1. **What is/are the most common method(s) of communication between the PBP and patients?**   **Please tick all that apply:** | | | | | | | | | | | | |
| --- | --- | --- | --- | --- | --- | --- | --- | --- | --- | --- | --- | --- |
| Email | Face-to-face | | | Fax | | Telephone | | Written | Other* | | Don’t know | |
| 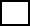 | 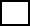 | | | 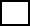 | | 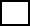 | | 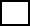 | 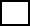 | | 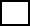 | |
| *Please specify below: | | | | | | | | | | | | |
| ___________________________________________________________________________________ | | | | | | | | | | | | |
| 1. **What is/are the most preferred method(s) of communication between the PBP and patients?**   **Please tick all that apply:** | | | | | | | | | | | | |
| Email | Face-to-face | | | Fax | | Telephone | | Written | Other* | | Don’t know | |
| 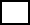 | 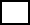 | | | 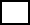 | | 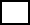 | | 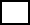 | 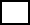 | | 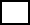 | |
| *Please specify below: | | | | | | | | | | | | |
| ___________________________________________________________________________________ | | | | | | | | | | | | |
| 1. **How often is a consulting room available for the PBP to use?** | | | | | | | | | | | | |
| Always | | | Very often | | Sometimes | | Rarely | | | Never | | |
| 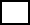 | | | 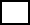 | | 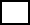 | | 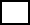 | | | 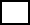 | | |
| 1. **What activities does the PBP undertake in your general practice?**   **Please tick all that apply:** | | | | | | | | | | | | |
| **Patient-level activities** | | - Medication reviews | | | | | | | | | | **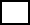** |
|  |  | - Medication reconciliation | | | | | | | | | | **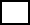** |
|  |  | - Triaging and managing minor ailments | | | | | | | | | | **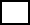** |
|  |  | - Counselling patients to help them manage their medications | | | | | | | | | | **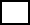** |
|  |  | - Educating patients on how to take their medicines | | | | | | | | | | **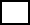** |
|  |  | - Educational group sessions to patients | | | | | | | | | | **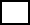** |
|  |  | - Lifestyle advice | | | | | | | | | | **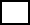** |
|  |  | - Addressing medicines adherence with patients | | | | | | | | | | **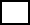** |
|  |  | - Acute prescribing | | | | | | | | | | **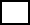** |
|  |  | - Repeat prescribing | | | | | | | | | | **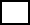** |
|  |  | - Signposting patients to appropriate services and other healthcare professionals (e.g. community pharmacists) | | | | | | | | | | **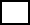** |
|  |  | - Managing other issues that involve medication such as adverse drug reactions and drug-drug interactions | | | | | | | | | | **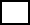** |
| **Health care provider-level activities** | | - Educational group sessions to health care providers | | | | | | | | | | **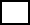** |
|  |  | - Answering medicines information enquiries from health care providers | | | | | | | | | | **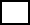** |
|  |  | - Student training | | | | | | | | | | **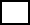** |
| **Practice/system-level activities** | | - Conducting clinical audits as part of the multidisciplinary team | | | | | | | | | | **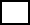** |
|  |  | - Administrative duties such as dealing with outpatient clinical letters and hospital discharge letters | | | | | | | | | | **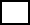** |
|  |  | - Developing clinical guidelines and/or practice formulary | | | | | | | | | | **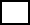** |
| **Other activities** | | - Research | | | | | | | | | | **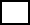** |
|  |  | - Outreach involvement (drug and therapeutics committee) | | | | | | | | | | **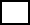** |
| **Other** | | - Please list any other activities undertaken by the PBP below: | | | | | | | | | |  |
| **___________________________________________________________________________________**  **___________________________________________________________________________________**  **___________________________________________________________________________________** | | | | | | | | | | | | |
| 1. **How were the PBP’s activities decided upon in your general practice?**   **Please tick all that apply:** | | | | | | | | | | | | |
| - Through mutual agreement between you and the PBP | | | | | | | | | | | | 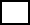 |
| - Determined by the PBP’s current skills | | | | | | | | | | | | 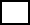 |
| - Determined by the PBP’s level of confidence | | | | | | | | | | | | 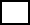 |
| - Determined by the PBP’s previous experience | | | | | | | | | | | | 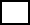 |
| - Other (please specify below): | | | | | | | | | | | | 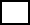 |
| ___________________________________________________________________________________ | | | | | | | | | | | | |
| 1. **Using the scale provided, please rate how often you encounter the following when dealing with the PBP, by placing a tick (**✓**) in the appropriate box:** | | | | | | | | | | | | |

|  | Always | Very often | Sometimes | Rarely | Never |
| --- | --- | --- | --- | --- | --- |
| - I do not have time to contact the PBP |  |  |  |  |  |
| - The PBP struggles to adapt to the needs of the practice |  |  |  |  |  |
| - The PBP has the clinical skills to provide safe and effective care for patients |  |  |  |  |  |
| - The PBP has the required experience to meet the needs of the practice |  |  |  |  |  |
| - The PBP is unavailable in the practice when I need them |  |  |  |  |  |
| - The PBP has the confidence to make clinical decisions |  |  |  |  |  |
| - The PBP has the knowledge to provide safe and effective care for patients |  |  |  |  |  |
| - Patients are reluctant to accept and book an appointment with the PBP |  |  |  |  |  |

**SECTION C: ATTITUDES TOWARDS COLLABORATION WITH PRACTICE-BASED PHARMACISTS**

*When answering the questions in this section, think of the PBP with whom you have had most dealings. Please indicate the extent to which you agree or disagree with the following statements by placing a tick (✓) in the appropriate box:*

|  | Strongly disagree | Disagree | Neither agree nor disagree | Agree | Strongly agree |
| --- | --- | --- | --- | --- | --- |
| 1. **The professional communication between myself and the PBP is open and honest** |  |  |  |  |  |
| 1. **The PBP is open to working together with me on patients’ medication management** |  |  |  |  |  |
| 1. **The PBP delivers high quality healthcare to patients** |  |  |  |  |  |
| 1. **The PBP has time to discuss matters with me relating to patients’ medication regimens** |  |  |  |  |  |
| 1. **The PBP meets the professional expectations I have of him/her** |  |  |  |  |  |
| 1. **I can trust the PBP’s professional decisions** |  |  |  |  |  |
| 1. **The PBP actively addresses patients' medical concerns** |  |  |  |  |  |
| 1. **The PBP and I have mutual respect for one another on a professional level** |  |  |  |  |  |
| 1. **The PBP and I share common goals and objectives when caring for the patient** |  |  |  |  |  |
| 1. **My role and the PBP’s role in patient care are clear** |  |  |  |  |  |
| 1. **I have confidence in the PBP's expertise in medicines and therapeutics** |  |  |  |  |  |
| 1. **The PBP has a role in assuring medication safety (for example, to identify drug interactions, adverse reactions, contraindications etc.)** |  |  |  |  |  |
| 1. **The PBP has a role in assuring medication effectiveness (for example, to ensure the patient receives the optimal drug at the optimal dose etc.)** |  |  |  |  |  |

**SECTION D: VIEWS OF PRACTICE-BASED PHARMACISTS AND THEIR IMPACT IN PRIMARY CARE**

*Please indicate the extent to which you agree or disagree with the following statements about practice-based pharmacists (PBPs) by placing a tick (*✓*) in the appropriate box:*

|  | Strongly disagree | Disagree | Neither agree nor disagree | Agree | Strongly agree |
| --- | --- | --- | --- | --- | --- |
| 1. **I welcome the PBP as part of the team** |  |  |  |  |  |
| 1. **The role of the PBP is clear to me** |  |  |  |  |  |
| 1. **I understand the difference between the roles of community pharmacists and PBPs.** |  |  |  |  |  |
| 1. **The introduction of the PBP role may take roles away from other members of the practice team** |  |  |  |  |  |
| 1. **The introduction of the PBP role moves community pharmacists to the periphery of the primary care team** |  |  |  |  |  |
| 1. **PBPs can provide a better link between general practices and community pharmacists** |  |  |  |  |  |
| 1. **The introduction of the PBP role will have a positive impact on patient outcomes** |  |  |  |  |  |
| 1. **PBPs will help in improving GPs’ knowledge and confidence about medications** |  |  |  |  |  |
| 1. **PBPs will help to alleviate pressure within primary care** |  |  |  |  |  |
| 1. **Having a PBP employed in general practices will save the NHS money by potentially freeing up GP time** |  |  |  |  |  |
| 1. **Having a PBP employed in general practices will save the NHS money by reducing medicine waste** |  |  |  |  |  |

1. **Do you have any further comments you wish to make about PBPs?**

_______________________________________________________________________________________________________________________________________________________________________________________________________________________________________________________________________________________________________________________________________________________________________________________

**Thank you for taking the time to complete this questionnaire; your response is much appreciated.**
