# Supplementary material for: General practitioners’ experiences with, views of, and attitudes towards, general practice-based pharmacists: a cross-sectional survey
Source: BMC Prim Care. 2022 Jan 14;23:6. doi: 10.1186/s12875-021-01607-5 (PMC8759266; doi:10.1186/s12875-021-01607-5)
Supplement: Supplementary file 3 — Additional file 3. Most common reasons for GP-PBP communication as reported by responding GPs. Description of data: Table summarising the most common reasons for GP-PBP communication (with selected examples) reported by GPs. [file 12875_2021_1607_MOESM3_ESM.docx]

**Additional file 3.** The most common reasons for GP-PBP communication as reported by responding GPs

| **Common reasons** | **Examples of common reasons for GPs to communicate with PBPs** | **Examples of common reasons for PBPs to communicate with GPs** |
| --- | --- | --- |
| Medication issues | Request advice on drug doses, side effects and drug interactions, etc. | To double check with GP about their decisions regarding medications |
| Medication review | Discussion of pharmacist-led reviews | Issues identified in medication reviews |
| Transitions between care sectors | Hospital letter (following up medication queries with hospital pharmacy/Drs) | To confirm if GP is happy with decisions or to authorize non-standard hospital advice |
| Prescribing queries | To request assistance with acute prescribing issues, e.g. finding unusual/infrequently prescribed items, and repeat dispensing | To verify and sign prescriptions generated by them |
| Patient issues | Asking pharmacist to follow-up with patient starting on new medication or complicated regimen stated hospital | Answering queries/questions regarding patients |
| Audit and COMPASS report^a^ | Results of audit work | |
| Projects and programmes | Feedback on projects and programmes such as quality improvement projects, drug withdrawal programmes, e.g. gabapentin, opiates | |
| Others | Regarding new guidelines and updating prescribing guidelines | |

^a^ Prescribing report issued quarterly for each GP practice
