# Supplementary material for: General practitioners’ experiences with, views of, and attitudes towards, general practice-based pharmacists: a cross-sectional survey
Source: BMC Prim Care. 2022 Jan 14;23:6. doi: 10.1186/s12875-021-01607-5 (PMC8759266; doi:10.1186/s12875-021-01607-5)
Supplement: Supplementary file 4 — Additional file 4. PBP-Patient communication as reported by responding GPs. Description of data: Three figures (A, B, and C) depicting PBP-Patient communication reported by GPs: Figure A shows frequency of face-to-face meetings between PBPs and patients; Figure B shows the most common method(s) of communication between PBPs and patients; and Figure C shows the most preferred method(s) of communication between PBPs and patients. [file 12875_2021_1607_MOESM4_ESM.docx]

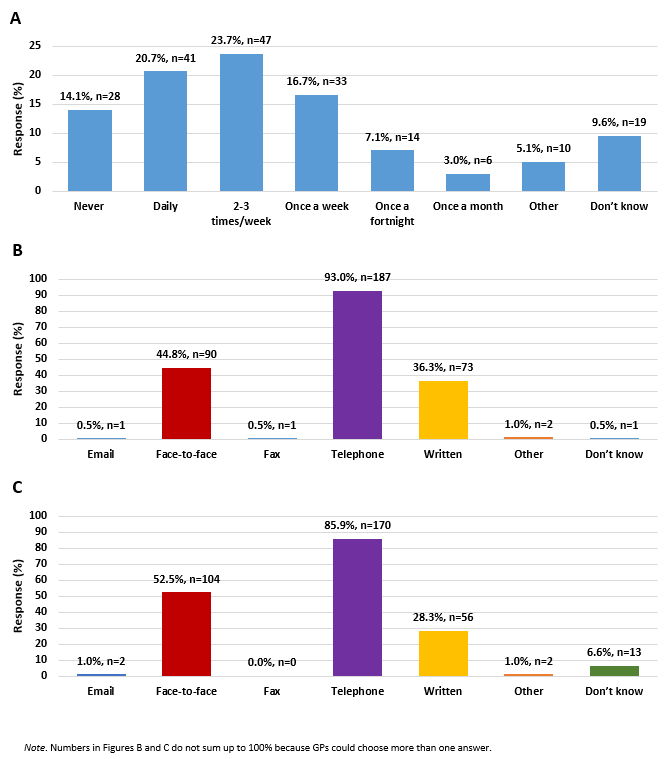
**Additional file 4.** PBP-Patient communication as reported by responding GPs

**Figure A. Frequency of face-to-face meetings between PBPs and patients.**

**Figure B. The most common method(s) of communication between PBPs and patients.**

**Figure C. The most preferred method(s) of communication between PBPs and patients.**
