# Supplementary figures and images for: General practitioners’ experiences with, views of, and attitudes towards, general practice-based pharmacists: a cross-sectional survey
Source: BMC Prim Care. 2022 Jan 14;23:6. doi: 10.1186/s12875-021-01607-5 (PMC8759266; doi:10.1186/s12875-021-01607-5)

**Additional file 5.** The frequency of availability of a consulting room for use by the PBP


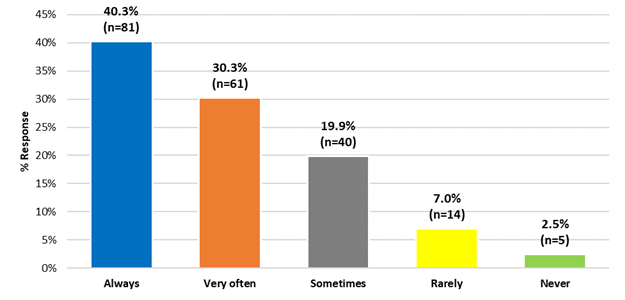

Supplement: Supplementary file 5 — Additional file 5. Frequency of availability of a consulting room for use by the PBP. Description of data: Figure showing the frequency of availability of a consulting room for use by the PBP as reported by GPs. [file 12875_2021_1607_MOESM5_ESM.docx]
