# Supplementary material for: General practitioners’ experiences with, views of, and attitudes towards, general practice-based pharmacists: a cross-sectional survey
Source: BMC Prim Care. 2022 Jan 14;23:6. doi: 10.1186/s12875-021-01607-5 (PMC8759266; doi:10.1186/s12875-021-01607-5)
Supplement: Supplementary file 6 — Additional file 6. Recommendations and issues for the PBP role with supporting comments reported by GPs. Description of data: Table summarising the recommendations and issues that have been reported by GPs in the free text comments with examples of GP quotes. [file 12875_2021_1607_MOESM6_ESM.docx]

| **Categorisation of free-text comments** | **GPs quotes** |
| --- | --- |
| More sessions | *“Excellent addition to primary care team. Need more than 3 sessions per week”* (GP136)  “*We only have 4 sessions a week so PBP not getting opportunity to run clinics, this needs urgently addressed*” (GP005)  *“…we would like more sessions” (GP189)* |
| Full time post | *“It would be better to have a full time PBP on site rather than job sharing”* (GP055)  *“Full time for all practices is vital and essential”* (GP093)  “*We need more PBPs. Need PBP available for all of the working week not just some sessions” (GP147)*  *“Every practice should have at least one full time PBP” (GP156)* |
| Working arrangements to be overseen by practices | *“Would like full time PBP and would like them to be under the control of the practice, and the practice holds the budget and employing the PBP directly”* (GP019)  “*It is very frustrating that PBPs are not employed by us. They work to an agenda driven by the Board via the Federation. We have problems with work plans and holidays*.” (GP052) |
| Lack of adequate cover | “*The PBP has been a very welcome addition to the practice but unfortunately, we are not getting the full benefit at the moment due to maternity leave and lack of adequate cover to replace the PBP during maternity leave”* (GP099)  *“The Federation in our area have unfortunately failed to provide adequate cover now that our PBP has gone on maternity leave. This has greatly impacted on the usefulness of PBP to our practice.”* (GP139) |
| Lack of adequate training | *“Need more training to upskill the pharmacists as independent prescribers”* (GP104)  *“They need more computer training specially around coding if possible before they start”* (GP111)  *“PBP need improved structure of training to suit GP, i.e. hypertension Diploma/vaccination training etc.”* (GP063)  “*Essential part of team. Need more training with other chronic diseases e.g. Diabetes/IHD*” (GP041) |
| Lack of independent prescribing qualification | *“…PBP needs her prescribing qualifications” (GP005)*  *“No independent prescribing, signing of prescription or patient clinical notes; their role very limited in my practice”* (GP145)  *“The current PBP is not a prescriber, and their role is limited by this.”* (GP090) |
| Lack of confidence | *“Our PBP is highly qualified but lacks the confidence to use these skills on a clinical basics”* (GP085)  *“Excellent resource and only marginally frees up GP time as requires input from us on regular basis. Perhaps will further improve with more confidence in their role”* (GP143)  *“The PBP experience in practices has been very much based on the individual PBPs. Some seem excellent, some are not confident and do not follow the agenda required by GP”* (GP151) |
| Lack of defined PBP role | “*Not enough time from pharmacist and job role not clearly defined. Could be doing a lot more*” (GP082)  *“Excellent having a PBP within the surgery. My only concern is the potential overlap with practice nurse. The role should focus more on alleviating GP time”* (GP058) |

**Additional file 6. Recommendations and issues for the PBP role with supporting comments reported by responding GPs from open-ended questions**
